# An unusual presentation of *UMOD*-associated autosomal dominant tubulointerstitial kidney disease in a pediatric patient

**DOI:** 10.1007/s00467-025-07024-5

**Published:** 2025-11-10

**Authors:** Andrew Murray, Monica Cramer

**Affiliations:** 1 Pediatrics, Prisma Health Children’s Hospital – Midlands/University of South Carolina School of Medicine – Columbia, Columbia, SC USA; 2 Pediatric Nephrology, Prisma Health Children’s Hospital – Midlands/University of South Carolina School of Medicine – Columbia, Columbia, SC USA

**Keywords:** Uromodulin, ADTKD, Hypertension, *UMOD*

## Abstract

Autosomal dominant tubulointerstitial kidney disease (ADTKD-*UMOD*) is a chronic and progressive kidney disease that typically presents with elevated serum creatinine and gout between the second and fourth decades of life. Our patient presented at age 6 years with persistent headaches and nosebleeds that began the year prior to presentation with nephrology. Stage 2 hypertension prompted her nephrology referral from the pediatrician. Laboratory evaluation was not initially remarkable for significant elevation in creatinine as would be expected; however, her creatinine level did eventually rise to a concerning level after months of antihypertensive therapy. Kidney biopsy was remarkable for findings consistent with *UMOD*-associated ADTKD. This case demonstrates an unusual presentation of ADTKD*-UMOD* with Stage 2 hypertension at an early age in the absence of significant kidney injury and highlights a further emerging role for genetic testing in suspected secondary hypertension.

## Case presentation

A 6-year-old female with a past medical history of anxiety disorder presented to the pediatric nephrology clinic for evaluation of elevated blood pressure. She reported symptoms of persistent headache and nosebleeds for the past 12 months, which the family attributed to a motor vehicle accident she sustained just prior to the initiation of symptoms. She was not noted to have any organ injury due to the motor vehicle accident. She denied any changes in vision, chest pain, palpitations, and nausea/vomiting. She had no recent illnesses and took no medications. She was previously healthy, was born full-term, and had no history of urinary tract infections. She had normal voiding patterns without enuresis. She denied hematuria, edema, joint pain, or rashes. She denied snoring. Dietary history included a typical pediatric diet for age with mostly home-cooked meals, occasional fast food, drinking mostly juice, and some water. There was no known family history of kidney disease. Physical examination was unremarkable (including growth parameters and vital signs for age) except for blood pressure of 124/80 (> 95th percentile + 12 systolic, > 95th percentile diastolic). A 24-h ambulatory blood pressure monitor confirmed hypertension with an average awake BP of 138/87 mmHg, an average asleep BP of 124/71 mmHg, and an average 24-h BP of 133/82 mmHg (all of the systolic values are >95th percentile for age) [1]. She began amlodipine as work-up was pursued.

Initial laboratory evaluation revealed normal electrolytes: creatinine 50.4 µmol/L (reference range 26.53–53.05 µmol/L), BUN 13 mg/dL. Urinalysis was unremarkable; specific gravity was 1.025. Thyroid stimulating hormone, renin, aldosterone, and serum metanephrines were all normal. An echocardiogram was normal. Kidney ultrasound was normal, both for kidney size and echogenicity. CT angiogram abdomen was negative for renal artery stenosis.

**Fig. 1:**
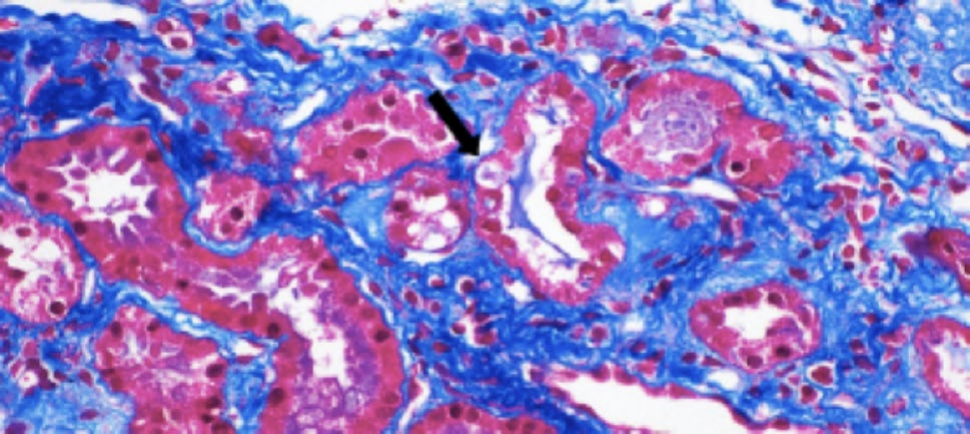
Kidney biopsy image of this patient (Arkana Laboratories). Light microscopy showing intra-cytoplasmic inclusions staining trichrome blue in the kidney tubular cells of the thick ascending limb (solid arrow)

During follow-up over the next several months, amlodipine was titrated and enalapril was added for ongoing hypertension. She was referred to genetics for evaluation of monogenic hypertension. Whole exome sequencing revealed no disease-causing variants, though she did have a heterozygous, non-maternal variant of undetermined clinical significance (VUS) in the *UMOD* gene. Nucleotide change c.155G > T. Amino acid change p.Cys52Phe. In silico prediction model was not provided by the testing laboratory. Mother was negative for the *UMOD* variant, and father’s screening is pending. Subsequent lab evaluations showed worsening creatinine to 0.86 mg/dL. A kidney biopsy was performed and revealed global glomerulosclerosis in 10/28 glomeruli, with moderate interstitial fibrosis and tubular atrophy. As seen in Fig. 1, cytoplasmic inclusions were present in thick ascending limbs of the loops of Henle (TALH). A uric acid level was 316 µmol/L (reference range, female 6–11 years; 142.8–333 µmol/L). No fractional excretion of uric acid (FeUA) was collected for the patient. Subsequent screening for autoimmune diseases, including lupus and sarcoidosis, was negative. Currently, her medication regimen is amlodipine 7.5 mg daily and lisinopril 20 mg daily. Her blood pressure in the office is 98/62 (50th percentile for age and height) [1]. Goal blood pressures are <90th percentile for age and height.

## Discussion

The mechanism underlying kidney injury progression in ADTKD-*UMOD* is misfolding of the uromodulin (UMOD) protein, which is encoded by the mutated *UMOD* gene. Uromodulin is a kidney-specific protein, expressed primarily in the thick ascending limb of Henle (TALH) and to a lesser degree in the distal convoluted tubule. Misfolded/mutated uromodulin cannot exit the endoplasmic reticulum (ER), causing abnormal deposition and accumulation. This causes ER stress, tubulointerstitial inflammation, accelerated apoptosis, tubular cell drop-out, and chronic kidney disease (CKD) due to interstitial fibrosis [2, 3].

A key feature of dysfunctional uromodulin is a decrease in the FeUA, leading to an increase in serum uric acid. This occurs as a downstream effect of sodium-coupled urate transporters within the proximal tubule, as they become scarred from uromodulin deposition. An additional mechanism of hyperuricemia is uromodulin retention within the endoplasmic reticulum of TALH cells, preventing uric acid excretion [4].

ADTKD-*UMOD* typically presents in patients in early adulthood with early onset gout and elevated creatinine levels (early onset CKD). These patients progress to kidney failure at a median age of 54 years. Cysts on kidney ultrasound are another common finding in these patients [5].

## Treatment/management

Treatment of ADTKD-*UMOD* is primarily symptomatic management. Aggressive blood pressure management is imperative, as hypertension will advance the chronic kidney disease process that has typically already begun at the time of presentation. While allopurinol may be beneficial to prevent attacks of gout, there is no evidence that it is beneficial in halting the progression of chronic kidney disease that is ongoing in patients with ADTKD-*UMOD.* There is some evidence for tumor necrosis factor-α inhibitors slowing disease progression, but this has not yet been definitively shown for long-term management [3].

## Summary

### What is new?

- This patient presented at an early age with Stage 2 hypertension without a significant elevation in creatinine or hyperuricemia, both of which are typically the first findings in adolescence.

## Data Availability

Data sharing is not applicable to this article as no datasets were generated or analyzed during the current study.
